# Causality between Celiac disease and kidney disease: A Mendelian Randomization Study

**DOI:** 10.1097/MD.0000000000039465

**Published:** 2024-08-30

**Authors:** Ya-mei Ge, Shuang-li Peng, Qiong Wang, Jun Yuan

**Affiliations:** a Clinical College of Chinese Medicine, Hubei University of Chinese Medicine, Hubei, Wuhan, China; b Department of Traditional Chinese Medicine, Renmin Hospital of Wuhan University, Hubei, Wuhan, China; c Department of Nephrology, Renmin Hospital of Wuhan University, Hubei, Wuhan, China; d First Clinical College, Hubei University of Chinese Medicine, Hubei, Wuhan, China.

**Keywords:** causal relationship, Celiac disease, genome-wide association studies, kidney disease, Mendelian Randomization Analysis

## Abstract

Celiac disease, characterized as an autoimmune disorder, possesses the capacity to affect multiple organs and systems. Earlier research has indicated an increased risk of kidney diseases associated with celiac disease. However, the potential causal relationship between genetic susceptibility to celiac disease and the risk of kidney diseases remains uncertain. We conducted Mendelian randomization analysis using nonoverlapping European population data, examining the link between celiac disease and 10 kidney traits in whole-genome association studies. We employed the inverse variance-weighted method to enhance statistical robustness, and results’ reliability was reinforced through rigorous sensitivity analysis. Mendelian randomization analysis revealed a genetic susceptibility of celiac disease to an increased risk of immunoglobulin A nephropathy (OR = 1.44; 95% confidence interval [CI] = 1.17–1.78; *P* = 5.7 × 10^−4^), chronic glomerulonephritis (OR = 1.15; 95% CI = 1.08–1.22; *P* = 2.58 × 10^−5^), and a decline in estimated glomerular filtration rate (beta = −0.001; *P* = 2.99 × 10^−4^). Additionally, a potential positive trend in the causal relationship between celiac disease and membranous nephropathy (OR = 1.37; 95% CI = 1.08–1.74; *P* = 0.01) was observed. Sensitivity analysis indicated the absence of pleiotropy. This study contributes novel evidence establishing a causal link between celiac disease and kidney traits, indicating a potential association between celiac disease and an elevated risk of kidney diseases. The findings provide fresh perspectives for advancing mechanistic and clinical research into kidney diseases associated with celiac disease.

## 1. Introduction

Kidney disease poses a worldwide public health challenge, impacting more than 750 million individuals globally.^[1]^ The global incidence of kidney disease is still rapidly increasing, and the incidence of end-stage renal disease (ESRD) necessitating dialysis or kidney transplantation is also on the rise.^[2]^ Diabetes, hypertension, and various forms of glomerulonephritis are the principal causative factors contributing to the development of ESRD.^[3]^ Globally, Immunoglobulin A nephropathy (IgAN) represents the most prevalent subtype among primary glomerulonephritis.^[4]^ Kidney disease significantly impacts extrarenal tissues, especially the cardiovascular system.^[5]^ This is also one of the reasons contributing to the mortality of patients with kidney disease. The global attention to kidney diseases is highly warranted not only due to their high prevalence and their coexistence with other common chronic conditions but also because of the substantial disease burden associated with kidney diseases. As the progression of kidney disease to ESRD requires maintenance dialysis therapy or kidney transplantation, kidney disease has transitioned from a subspecialty condition to a worldwide public health concern. Considering the substantial impact on human health and the associated economic implications of kidney disease, early screening and preventive measures for kidney disease become significantly crucial.

Celiac disease is a systemic condition marked by immune-mediated damage to the small intestine, triggered by the consumption of gluten in individuals genetically predisposed to the disorder. Celiac disease is widely distributed worldwide, with a prevalence in the general population ranges from 0.5% to 2%, with an average of about 1%.^[6]^ Furthermore, patients with celiac disease may present with typical gastrointestinal symptoms or atypical extraintestinal manifestations. Consequently, a significant number of individuals with celiac disease go undetected, receive incorrect diagnoses, or encounter considerable delays in being diagnosed.^[7]^ Celiac disease is often accompanied by various extraintestinal manifestations, any organ may be involved due to autoimmune/ inflammatory responses or malabsorption.^[8]^

To date, an accumulation of evidence has indicated potential associations between celiac disease and kidney disease risk.^[9,10]^ A Swedish cohort study involving 29,050 patients indicates that individuals with celiac disease face an elevated risk of developing ESRD (risk ratio = 2.47; 95% CI = 1.80–3.40).^[11]^ A recent study based on the European population indicated that the risk of having any glomerulonephritis was threefold in patients with celiac disease compared to that of reference individuals, while the elevated risk of IgAN was over 18-fold.^[10]^

However, observational studies are prone to bias due to confounding factors and reverse causation. Mendelian randomization (MR) utilizes genetic variations associated with modifiable exposures (or risk factors) to assess potential causal relationships with outcomes.^[12]^ This approach leverages inherent genetic variations present in individuals from birth, typically independent of confounding factors and biases, thereby reducing the occurrence of bias.^[13]^ Therefore, this method provides more reliable evidence. Currently, no MR analysis has confirmed a causal relationship between celiac disease and kidney disease. This study aims to examine the association between celiac disease and the risk of kidney diseases using a 2-sample MR approach. To achieve this, we utilized a large-scale GWAS database and data from published literature to assess the causal relationships between celiac disease and chronic kidney disease (CKD), IgAN, membranous nephropathy (MN), diabetic nephropathy (DN), nephrotic syndrome (NS), glomerulonephritis, urolithiasis, estimated glomerular filtration rate (eGFR), and urine albumin-to-creatinine ratio (UACR).

## 2. Materials and methods

### 2.1. Data source for kidney disease and celiac disease

In order to address the potential impact of population stratification, we specifically obtained single nucleotide polymorphisms (SNPs) and their associated summary data from studies conducted exclusively with populations of European ancestry. Summary-level data for CKD and eGFR were derived from a meta-analysis conducted by the CKD Genetics Consortium, encompassing 41,395 cases and 439,303 controls.^[14]^ Meta-analysis data for UACR were retrieved from CKD genetics, comprising 51,861 cases and 297,093 controls.^[15]^ Summary-level statistics for primary MN were extracted from a meta-analysis of GWAS, including data from 2150 cases and 5829 control subjects.^[16]^ Summary statistics for IgAN were acquired from the FinnGen research database (https://r9.finngen.fi/), comprising 863 cases and 7682 controls. GWAS summary statistics for DN, urolithiasis, chronic or acute glomerulonephritis, and NS were retrieved from the IEU GWAS datasets (https://gwas.mrcieu.ac.uk/). We obtained publicly available GWAS data for celiac disease from the IEU GWAS database. This study included data from 12,041 cases of celiac disease and 12,228 controls.^[17]^ To validate the precision of our findings, we employed an additional GWAS dataset on celiac disease (https://gwas.mrcieu.ac.uk/datasets/finn-b-K11_COELIAC/). This study encompassed 1973 celiac disease patients and 210,964 control cases.

### 2.2. Selection of IVs

In MR analysis, SNPs strongly linked to the risk factor can be employed to investigate the causal impact of the risk factor on an outcome. SNPs meeting the 3 fundamental assumptions can be chosen as genetic instrumental variables (IVs) for implementation in MR analysis. First, the chosen IVs must exhibit a robust association with celiac disease. Second, the IVs should be independent of confounding factors that affect both the celiac disease and kidney traits. Third, the chosen IVs ought to influence outcomes solely through the celiac disease.^[12]^

To avoid weak-instrument bias, we obtained the genome-wide significant variants (*P* < 5 × 10^−8^) reliably associated with celiac disease to serve as SNPs for IVs^[18]^ and then computed the F-statistic for each SNP. In the MR model, the strength of IVs is typically assessed through the F-statistic.^[19]^ An empirical rule of thumb is that the F-statistic for IV strength in the MR model should be at least 10.

**Figure 1.**
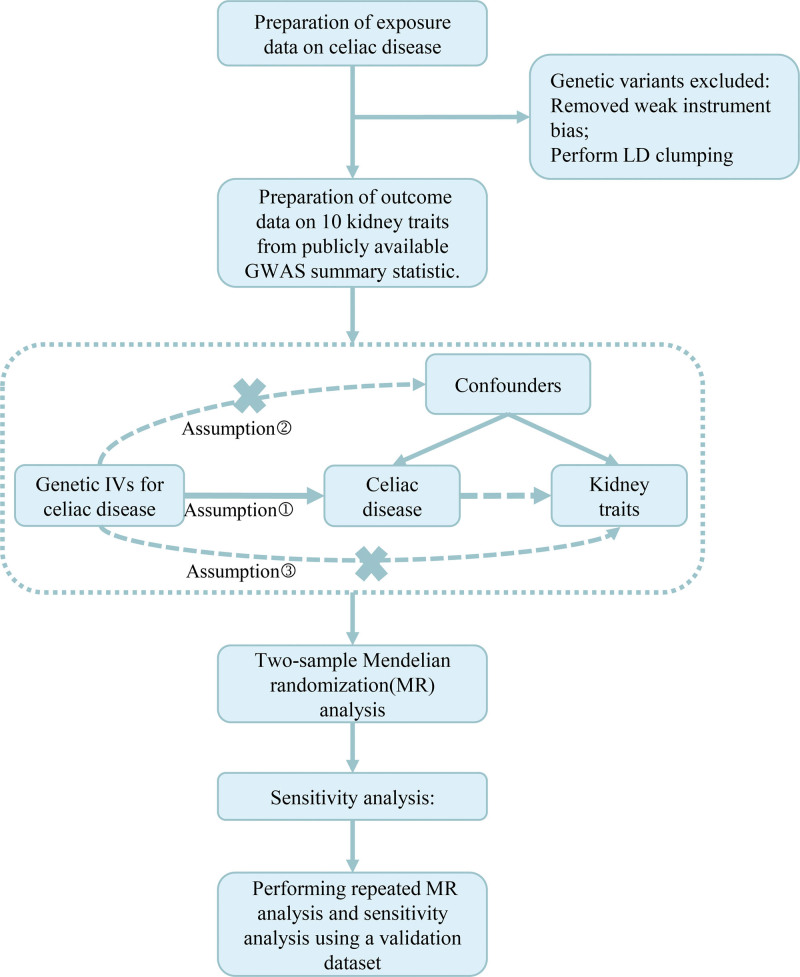
Flowchart. GWAS = whole-genome association studies, IV = instrumental variable, LD = linkage disequilibrium, MR = Mendelian Randomization.

To address potential bias stemming from significant linkage disequilibrium among the selected IVs, the linkage disequilibrium parameters were set as follows: r² < 0.001 and a clumping distance of 10,000 base pairs (kb). Subsequently, we uploaded SNPs related to celiac disease and kidney diseases to the LDlink website (https://ldlink.nih.gov/?tab=home) and excluded SNPs associated with confounding factors. The research design and process are shown in Figure 1.

### 2.3. Statistical analyses

We applied a 2-sample MR analysis method to investigate the causal relationship between celiac disease and various kidney disorders. The primary analytical approach in the MR analysis was the inverse variance weighting (IVW) method, which was employed consistently across different models.^[20]^ Additionally, we concurrently incorporated 4 algorithms—MR Egger, weighted median, simple mode, and weighted mode—to assess the causal relationship between celiac disease and kidney diseases. Bonferroni correction was applied to adjust for multiple testing (An effect size is deemed significantly positive when its *P* value <0.005 [0.05/10], and considered potentially positive when its *P* value falls between 0.005 and 0.05).^[21]^ Statistical analyses were conducted using R software (version 4.3.1) and involved the utilization of the “TwoSampleMR” and “MR-PRESSO” R packages.

Cochran’s Q statistic was employed to evaluate the heterogeneity among the included IVs. A *P* value < 0.05 was considered indicative of significant heterogeneity. Subsequently, we utilized the MR-Egger intercept method to investigate the presence of horizontal pleiotropy in IVs,^[22]^ where a *P* value < 0.05 indicated pleiotropy. Additionally, we applied the MR-PRESSO method to identify and remove SNPs with notable differences, aiming to mitigate horizontal pleiotropy in the analysis.^[23]^ We conducted a leave-one-out sensitivity analysis to confirm the impact of abnormal IVs on the causal effect estimates, ensuring the robustness and stability of the results.^[24]^ Finally, we generated funnel plots, scatter plots, and forest plots.

## 3. Results

After eliminating SNPs with linkage disequilibrium, low association strength, and palindromic sequences, 29 SNPs remained as candidate IVs for celiac disease. The final inclusion of SNPs was adjusted based on confounding factors associated with different kidney diseases and the results of the MR-PRESSO outlier test. Comprehensive details regarding the genetic variants are presented in Table S1, Supplemental Digital Content, http://links.lww.com/MD/N453. No indications of weak-instrument bias were detected in the IVs strength test (F-statistic > 10).

**Figure 2.**
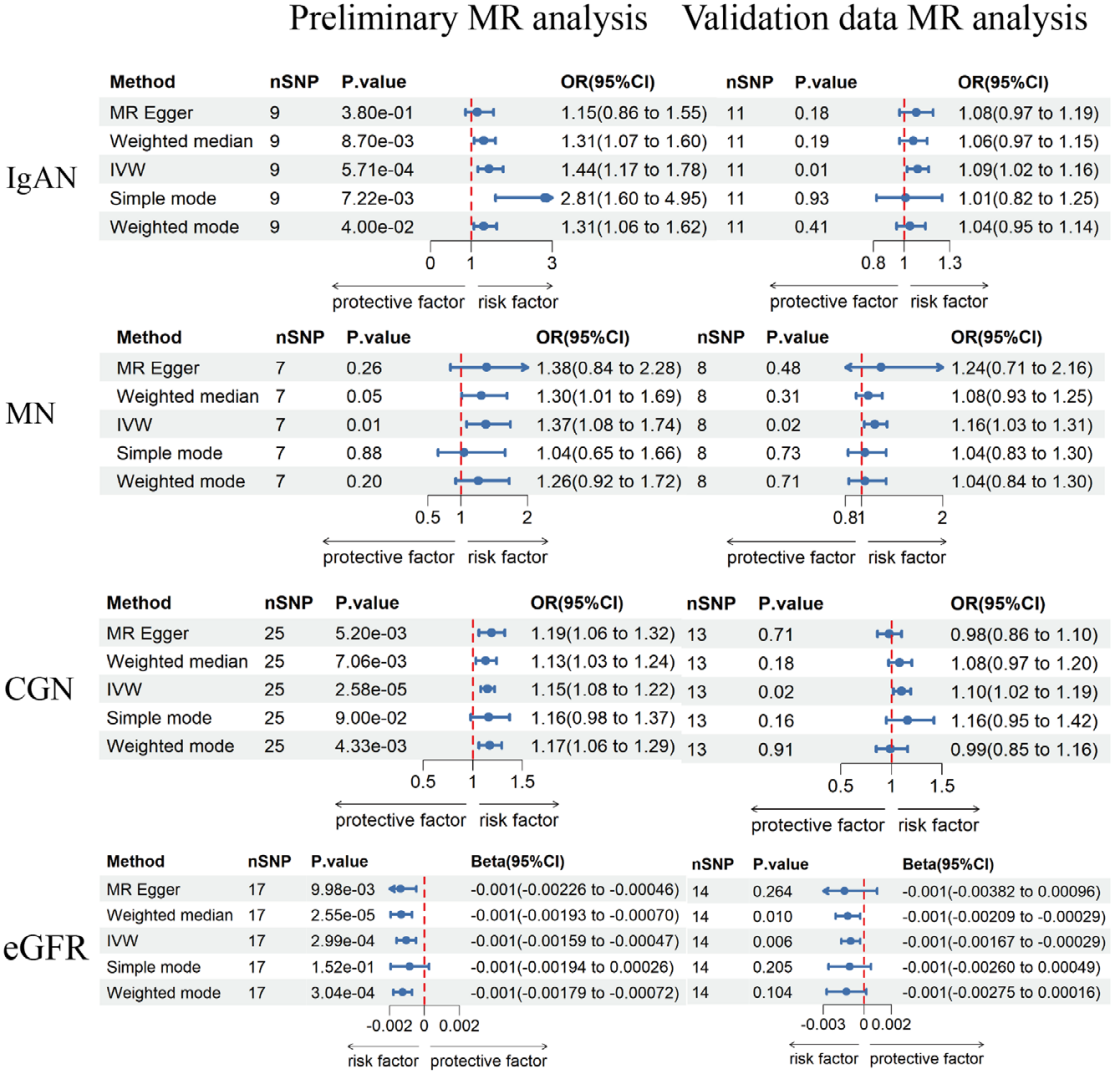
MR estimates of celiac disease on the risk for IgAN, MN, CGN, and eGFR. CGN = chronic glomerulonephritis, eGFR = estimated glomerular filtration rate, IgAN = IgA nephropathy, IVW = inverse variance-weighted, MN = membranous nephropathy.

**Figure 3.**
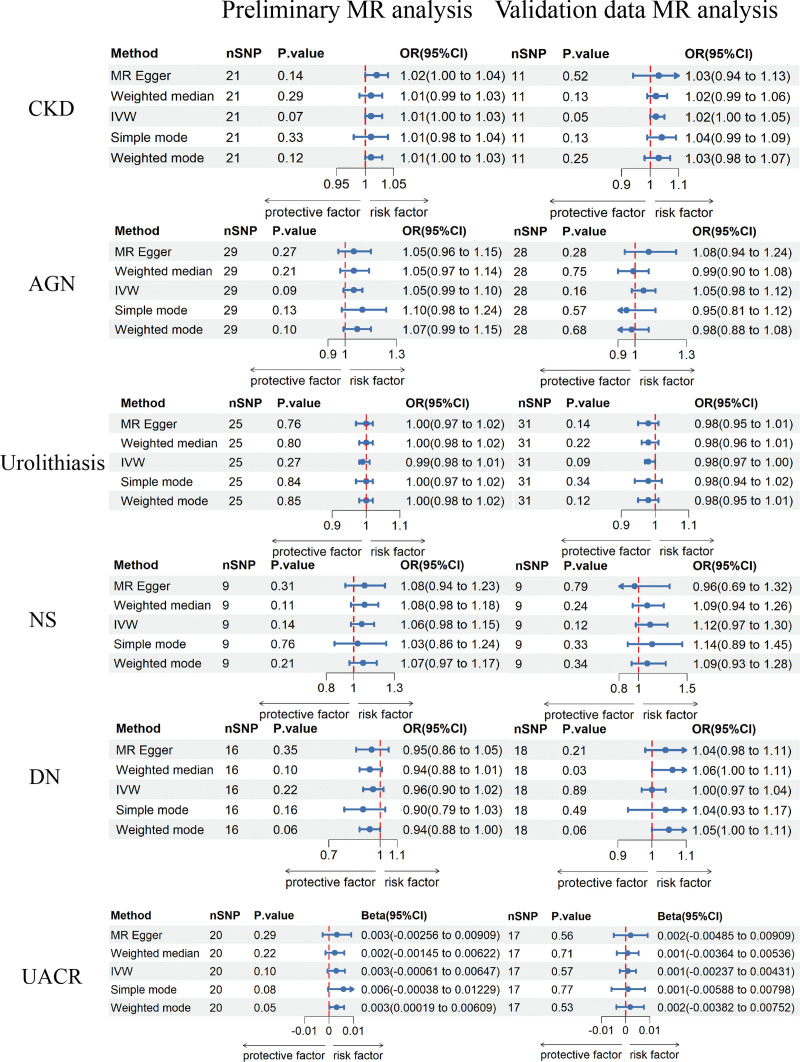
MR estimates of celiac disease on the risk for CKD, AGN, urolithiasis, NS, DN, and UACR. AGN = acute glomerulonephritis, CKD = chronic kidney disease, DN = diabetic nephropathy, IVW = inverse variance-weighted, NS = nephrotic syndrome, UACR = urine albumin-to-creatinine ratio.

The IVW method revealed a genetic susceptibility of celiac disease to an increased risk of IgA nephropathy (OR = 1.44; 95% CI = 1.17–1.78; *P* = 5.7 × 10^−4^), chronic glomerulonephritis (OR = 1.15; 95% CI = 1.08–1.22; *P* = 2.58 × 10^−5^), MN (OR = 1.37; 95% CI = 1.08–1.74; *P* = .01), and a decline in eGFR (beta = −0.001; *P* = 2.99 × 10^−4^). After Bonferroni correction, significant causal associations were found between celiac disease and IgAN, chronic glomerulonephritis, and eGFR. The causal relationship between celiac disease and MN exhibited a potential positive trend. However, the prevalence differences for CKD (OR = 1.01; 95% CI = 1.00–1.03; *P* = .07), acute glomerulonephritis (OR = 1.05; 95% CI = 0.99–1.10; *P* = .09), urolithiasis (OR = 0.99; 95% CI = 0.98–1.01; *P* = .27), NS (OR = 1.06; 95% CI = 0.98–1.15; *P* = .14), and DN (OR = 0.96; 95% CI = 0.90–1.02; *P* = .22) did not show statistical significance. Furthermore, celiac disease showed no statistically significant impact on the changes in UACR (beta = 0.003; *P* =.10). The results of the MR analysis are shown in Figures 2 and 3.

The leave-one-out method indicated that individual SNPs did not exert influence on the potential causal association between celiac disease and kidney diseases (Figure S1, Supplemental Digital Content, http://links.lww.com/MD/N454). Additionally, the MR-Egger regression intercept suggested limited evidence of pleiotropy in the IVs associated with Celiac disease and kidney diseases. The MR-PRESSO method ultimately revealed no outliers causing pleiotropic effects in the causal relationship between celiac disease and kidney diseases. All MR-PRESSO Global Test *P* values exceeded 0.05. In the heterogeneity tests, both the IVW and MR-Egger methods indicated that there was no heterogeneity (*P* > .05) among the IVs in the MR analyses of celiac disease and kidney diseases (CKD, IgAN, MN, urolithiasis, acute glomerulonephritis, chronic glomerulonephritis, NS, DN, and eGFR). However, there was heterogeneity (*P* = .03) among the IVs in the MR analysis of celiac disease and UACR. The results of sensitivity analyses and heterogeneity analysis are presented in Table 1. The scatter plots illustrate that as the risk of celiac disease increases, the risks of IgAN, chronic glomerulonephritis, and MN gradually increase, while eGFR gradually decreases. The directions of the results are consistent across the 3 methods, indicating stable results (Figure S2, Supplemental Digital Content, http://links.lww.com/MD/N454). Furthermore, the funnel plot and forest plot also indicate that the results of the MR analysis are relatively stable (Figures S3 and S4, Supplemental Digital Content, http://links.lww.com/MD/N454).

**Table 1:** Pleiotropy and heterogeneity test of the Celiac disease IVs from kidney traits GWAS.

Outcomes	Pleiotropy test			Heterogeneity test	
	MR-Egger		MR-PRESSO	MR Egger	Inverse variance weighted
	Intercept	*P*value	Global Test P	Q_pval	Q_pval
IgA nephropathy	0.0775	0.10	0.35	0.42	0.22
Chronic glomerulonephritis	−0.0194	0.46	0.21	0.22	0.24
Membranous nephropathy	−0.0494	0.41	0.20	0.07	0.07
eGFR	0.0002	0.38	0.09	0.06	0.05
Chronic kidney disease	−0.0021	0.60	0.60	0.51	0.55
Urolithiasis	−0.0023	0.69	0.18	0.17	0.20
Acute glomerulonephritis	−0.0025	0.91	0.62	0.59	0.64
Diabetic nephropathy	0.0045	0.78	0.16	0.11	0.14
Nephrotic syndrome	−0.0057	0.81	0.76	0.62	0.71
UACR	−0.0002	0.89	0.06	0.03	0.04

eGFR = estimated glomerular filtration rate, Q = heterogeneity statistic Q, UACR = urine albumin-to-creatinine ratio.

The results of the validated data aligned with the previously discussed findings with distinctions in the degree of risk and the significance of the outcomes. After excluding weak instrument variables and addressing linkage disequilibrium, the corresponding IVs were incorporated into the MR analysis. Comprehensive details regarding the IVs were presented in Table S2, Supplemental Digital Content, http://links.lww.com/MD/N453. The results indicated that the IVW method uncovered a link between celiac disease and an increased risk of IgAN (OR = 1.09; 95% CI = 1.02–1.15; *P* = .01), MN (OR = 1.16; 95% CI = 1.03–1.31; *P* = .02), chronic glomerulonephritis (OR = 1.10; 95% CI = 1.02–1.19; *P* = .02), and an increased risk of decreased eGFR (beta = −0.001; *P* = .006). The prevalence differences for CKD (OR = 1.02; 95% CI = 1.00–1.05; *P* = .05), acute glomerulonephritis (OR = 1.05; 95% CI = 0.98–1.12; *P* = .16), urolithiasis (OR = 0.98; 95% CI = 0.97–1.00; *P* = .09), NS (OR = 1.12; 95% CI = 0.97–1.30; *P* = .12), and DN (OR = 1.00; 95% CI = 0.97–1.04; *P* = .89) did not show statistical significance. Celiac disease showed no statistically significant impact on the changes in UACR (beta = 0.001; *P* = .57). The MR analysis results of the validation data were shown in Figures 2 and 3. The leave-one-out method indicated that individual SNPs did not exert influence on the potential causal association between celiac disease and kidney diseases (Figure S7, Supplemental Digital Content; http://links.lww.com/MD/N454). During the heterogeneity test, both the IVW and MR-Egger methods did not provide any evidence of heterogeneity (heterogeneity test *P* > .05) in the causal association between Celiac Disease and various kidney traits (Table S3, Supplemental Digital Content; http://links.lww.com/MD/N453). The regression intercept in the MR-Egger analysis, along with the MR-PRESSO method, both indicated the absence of pleiotropy (Table S3, Supplemental Digital Content, http://links.lww.com/MD/N453). Scatter plots, funnel plots, and forest plots, which offered a more intuitive representation of heterogeneity (Figures S5, S6, and S8, Supplemental Digital Content, http://links.lww.com/MD/N454).

## 4. Discussion

MR was utilized for the initial exploration, systematically examining potential causal relationships between susceptibility to celiac disease and the risk of kidney diseases. The results of this study suggested that a genetic predisposition to celiac disease was associated with an increased risk of IgAN, chronic glomerulonephritis, and a reduced level of eGFR. Furthermore, there appeared to be a potential positive trend in the causal relationship between celiac disease and MN.

The prevalence of celiac disease, an autoimmune disorder affecting genetically susceptible individuals, is increasing. Its occurrence is linked to mechanisms encompassing increased intestinal permeability, human leukocyte antigen recognition, and disease-specific autoantibody response to gluten peptides, targeting autoantigens like tissue transglutaminase 2 (TG2).^[8]^ While the intestinal symptoms of this disease are most pronounced, its impact extends to organs throughout the body. This could be attributed to the deposition of gluten-dependent circulating antibodies targeting TG2 in extraintestinal tissues.^[25]^ Abundant findings from observational studies suggest a heightened risk of kidney disease among individuals with celiac disease. Our study also indicates a positive correlation between celiac disease and a decline in eGFR. A cohort study conducted in the general population reveals that patients with celiac disease carry a 1.64 times higher risk of developing any glomerulonephritis compared to the reference population.^[26]^ Our research findings align with this trend of previous studies. However, our study further indicates an increased association between celiac disease and the risk of chronic glomerulonephritis, while it shows no correlation with the risk of acute glomerulonephritis.

A proposed mechanism suggests that in individuals with celiac disease, exposure to gluten proteins activates the mucosal immune system, triggering immune responses that target antigens in the gut. These responses subsequently cross-react with the glomerulus.^[27,28]^ Inflammation response and disruption of the intestinal barrier are also implicated in this process.^[29]^ Reportedly, the intestinal barrier damage in celiac disease closely resembles that in IgAN patients.^[30,31]^ Some studies suggest a higher prevalence of IgA nephropathy in patients with celiac disease, while others indicate the opposite. In a prospective study, individuals with celiac disease were found to face a 3-fold increased risk of future IgAN.^[32]^ Researchers have identified deposits of IgA targeting TG2 in renal tissue biopsies of IgAN patients, both with and without coexisting celiac disease.^[33]^ In a Finnish study, 827 patients who underwent kidney biopsies were tested for serum IgA tissue transglutaminase antibodies, revealing that 4.5% of overall kidney disease cases exhibited tissue transglutaminase antibodies. Among IgAN patients, this figure increased to 8.2%, and those with positive antibodies exhibited poorer kidney function.^[30]^ Research indicated that approximately 12% to 22% of IgAN patients tested positive for anti-gliadin IgA antibodies.^[34,35]^ These findings suggest an association between celiac disease and IgAN. However, an American study with 295 participants revealed that TG2 levels in IgA nephropathy patients did not exhibit a significant difference when compared to the healthy control group.^[36]^ Other research has also indicated that non-IgA nephropathy (minimal change disease, membranous glomerulonephritis, membranoproliferative glomerulonephritis) may exhibit positive immunohistochemical staining for anti-TG2, and the scoring is nearly comparable to that of IgAN.^[37]^ Another study also demonstrated that deposits of IgA antibodies against TG2 were present in the kidney tissues of both IgAN and non-IgAN patients. Such deposits were absent in normal renal tissues.^[38]^ The conclusions of earlier studies remain contentious; however, our research indicates an increased risk of IgAN in individuals with celiac disease.

Previous research indicates that immune reactions occurring in patients with celiac disease upon exposure to gluten may not only contribute to the development of IgAN but also potentially lead to other kidney disorders.^[39]^ Partial observational studies suggest an elevated risk of DN in individuals with type 1 diabetes who also have coexisting celiac disease.^[10,40–42]^ A retrospective analysis of 5533 participants in Sweden revealed that individuals with both type 1 diabetes and concurrent celiac disease face a 1.43 times higher risk of developing CKD compared to those with only type 1 diabetes.^[43]^ A meta-analysis indicated that patients with celiac disease had a 1.49 times increased risk of DN.^[9]^ Clinical studies have indicated that a gluten-free diet possibly has favorable effects on the development of vascular complications in patients with type 1 diabetes mellitus.^[44,45]^ However, a Danish study found no association between celiac disease and the onset of DN in type 1 diabetes patients.^[46]^ A prospective study in the United States identified an association between the concurrent presence of celiac disease or elevated serum TG2 titers and a decreased risk of kidney disease and significant albuminuria in individuals with type 1 diabetes.^[47]^ The conclusions of previous studies have been contentious. Our study indicates that there is no significant correlation between celiac disease and an increased risk of DN or UACR. Previous studies were primarily based on patients with type 1 diabetes, while our research data did not differentiate between type 1 DN and type 2 DN; instead, the data were aggregated, which might introduce some bias.

In previous studies, the correlation between celiac disease and MN was infrequently observed. Only a handful of case reports have documented instances where MN developed subsequent to the diagnosis of celiac disease, with other potential causes systematically ruled out. Improvement in the condition was noted upon adopting a gluten-free diet.^[48,49]^ Research has highlighted the presence of characteristic TG2 antibodies associated with celiac disease in the kidney tissues of MN patients.^[37]^ Another study identified a significant increase in T lymphocytes in the small intestine epithelium of individuals with MN.^[50]^ Our study identified a potential causal relationship between celiac disease and MN.

Our study, for the first time, examined the potential causal relationship between celiac disease and kidney traits. Previous observational and clinical studies have yielded conflicting conclusions concerning the causal connection between celiac disease and kidney disease. Our research, employing the MR method from a genetic epidemiological standpoint, has made a contribution to elucidating this matter. We utilized various methodologies to attain consistent conclusions, mitigating issues of heterogeneity and pleiotropy.

Simultaneously, our study had some limitations. GWAS results may vary across populations, and our study utilized GWAS data specific to the European population. Therefore, extrapolating the causal relationship between celiac disease and kidney disease to other populations is challenging. Additionally, our study examining the association between celiac disease and DN utilized GWAS data for DN in general, rather than specific data for type 1 DN.

## 5. Conclusions

Our findings indicated a genetic predisposition to celiac disease associated with an increased risk of IgAN, chronic glomerulonephritis, and a decline in eGFR. Additionally, a potential positive trend in the causal relationship between celiac disease and MN was observed. The study has enhanced our understanding of the mechanisms underlying the development of kidney diseases in individuals with celiac disease and lays the groundwork for evaluating the risk of kidney diseases in celiac disease patients and reducing the incidence of kidney diseases in a clinical setting.

## Acknowledgments

We express gratitude to all participants and researchers for their engagement in this MR study, as well as to all contributors to the GWAS database.

## Author contributions

**Conceptualization:** Ya-mei Ge.

**Methodology:** Ya-mei Ge.

**Validation:** Ya-mei Ge, Shuang-li Peng, Qiong Wang.

**Writing – original draft:** Ya-mei Ge.

**Software:** Shuang-li Peng.

**Data curation:** Qiong Wang.

**Project administration:** Jun Yuan.

**Supervision:** Jun Yuan.

**Writing – review & editing:** Jun Yuan.

## Supplementary Material


