# Supplementary material for: Causality between Celiac disease and kidney disease: A Mendelian Randomization Study
Source: Medicine (Baltimore). 2024 Aug 30;103(35):e39465. doi: 10.1097/MD.0000000000039465 (PMC11365674; doi:10.1097/MD.0000000000039465)
Supplement: Supplementary file 2 [file medi-103-e39465-s002.pdf]

Supplementary Figure S1

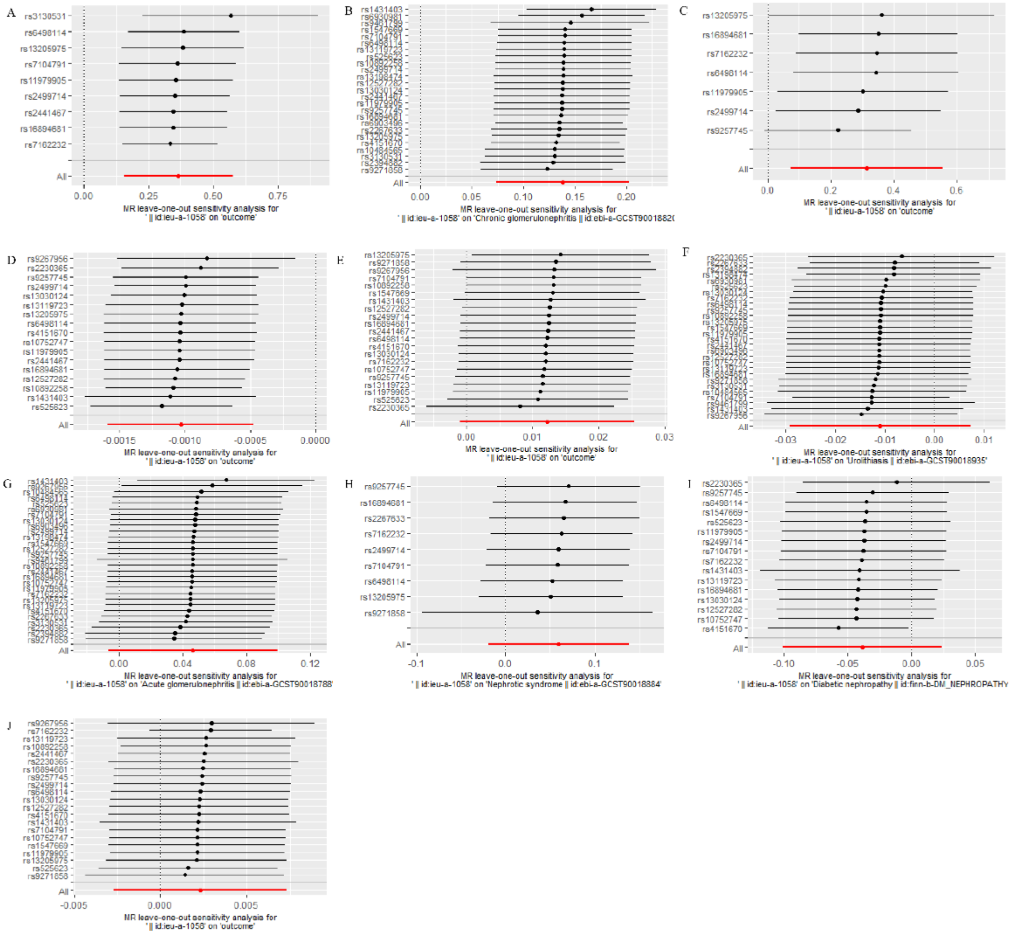

Figure S1 Leave-one-out sensitivity analysis: (A) Celiac disease and IgAN; (B) Celiac disease and Chronic glomerulonephritis; (C) Celiac disease and MN; (D) Celiac disease and eGFR; (E) Celiac disease and CKD; (F) Celiac disease and Urolithiasis; (G) Celiac disease and Acute glomerulonephritis;(H) Celiac disease and NS;(I) Celiac disease and DN;(J) Celiac disease and UACR.

Supplementary Figure S2

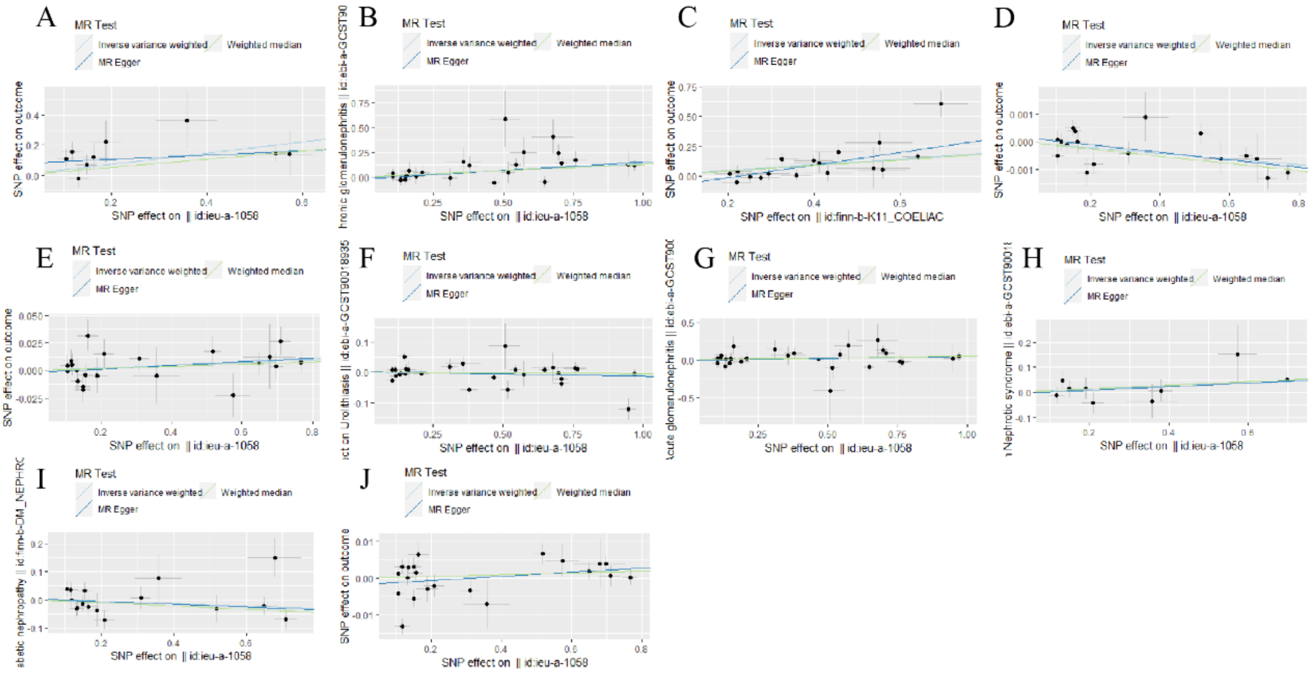

Figure S2 Scatter plot: (A) Celiac disease and IgAN; (B) Celiac disease and Chronic glomerulonephritis; (C) Celiac disease and MN; (D) Celiac disease and eGFR; (E) Celiac disease and CKD; (F) Celiac disease and Urolithiasis; (G) Celiac disease and Acute glomerulonephritis;(H) Celiac disease and NS;(I) Celiac disease and DN;(J) Celiac disease and UACR.

Supplementary Figure S3

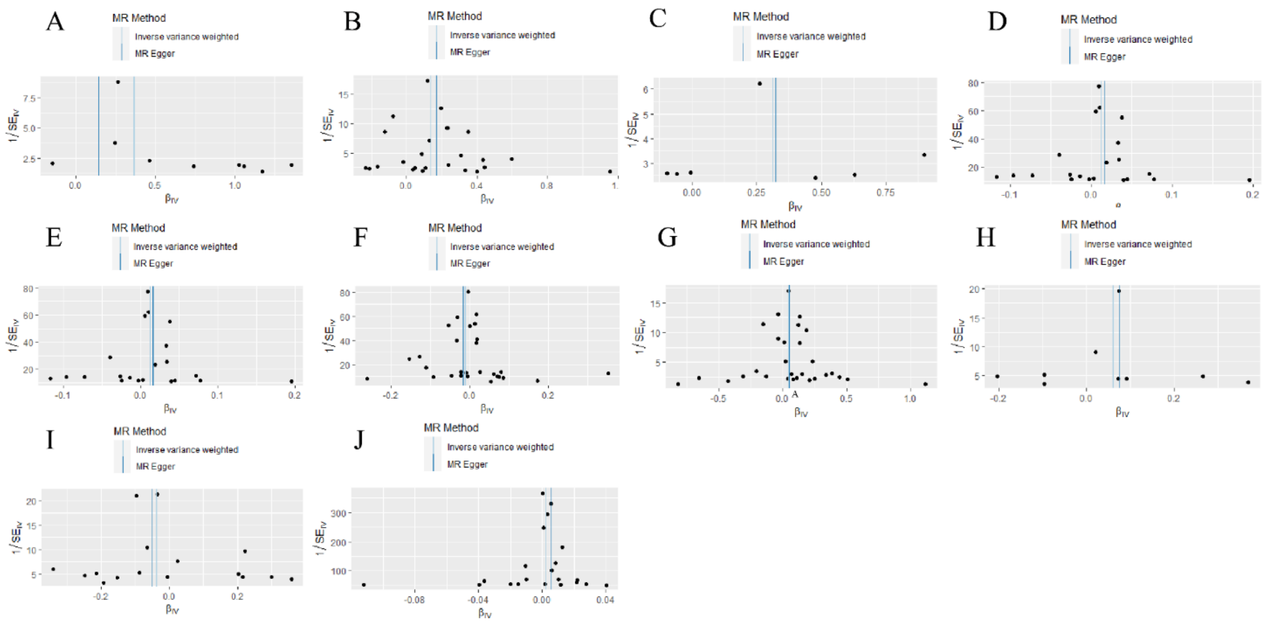

Figure S3 Funnel plot: (A) Celiac disease and IgAN; (B) Celiac disease and Chronic glomerulonephritis; (C) Celiac disease and MN; (D) Celiac disease and eGFR; (E) Celiac disease and CKD; (F) Celiac disease and Urolithiasis; (G) Celiac disease and Acute glomerulonephritis;(H) Celiac disease and NS;(I) Celiac disease and DN;(J) Celiac disease and UACR.

Supplementary Figure S4

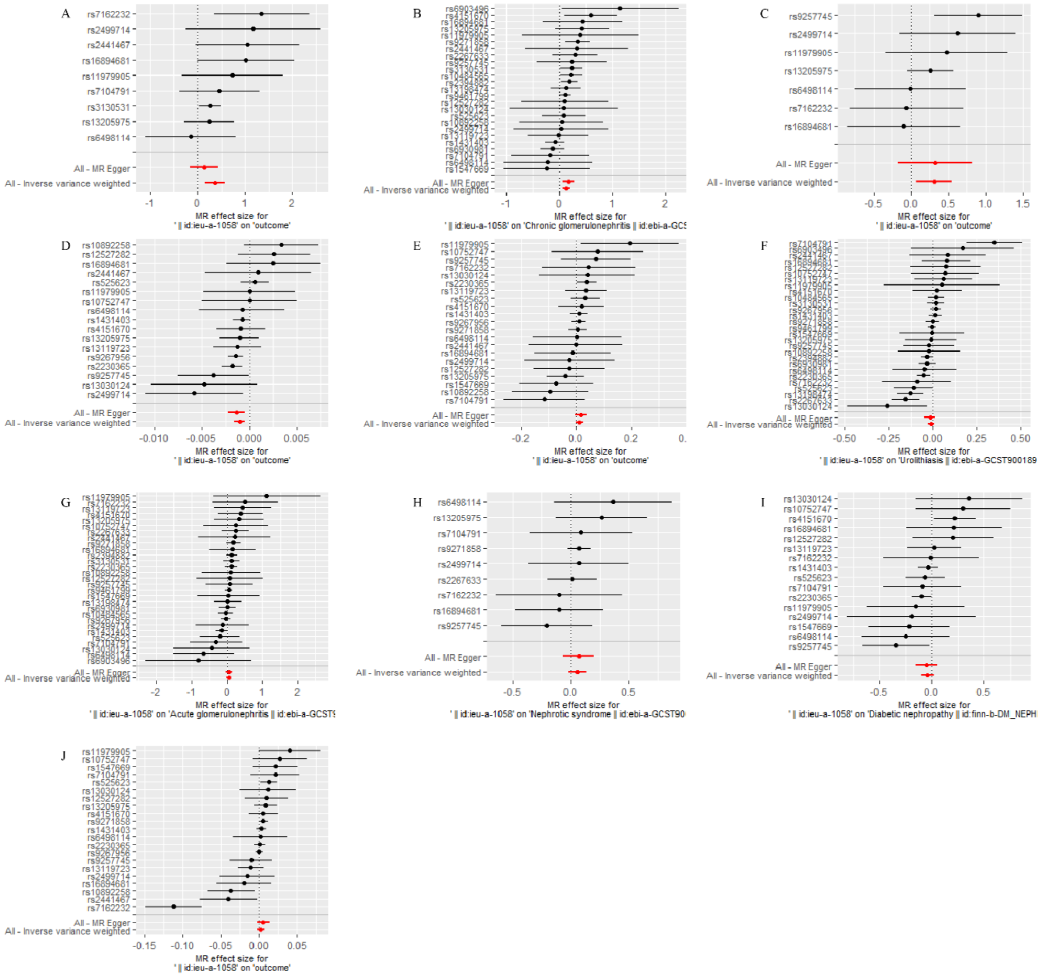

Figure S4 Forest plot: (A) Celiac disease and IgAN; (B) Celiac disease and Chronic glomerulonephritis; (C) Celiac disease and MN; (D) Celiac disease and eGFR; (E) Celiac disease and CKD; (F) Celiac disease and Urolithiasis; (G) Celiac disease and Acute glomerulonephritis;(H) Celiac disease and NS;(I) Celiac disease and DN;(J) Celiac disease and UACR.

Supplementary Figure S5

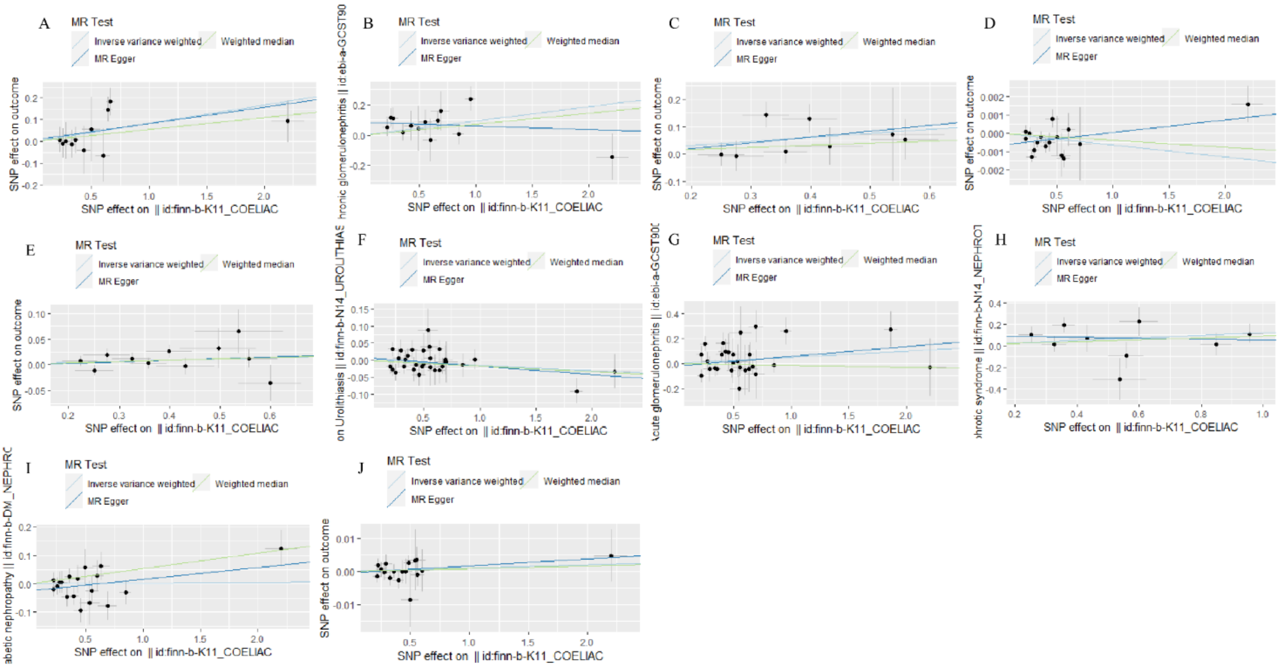

Figure S5 Validation data MR analysis scatter plot: (A) Celiac disease and IgAN; (B) Celiac disease and Chronic glomerulonephritis; (C) Celiac disease and MN; (D) Celiac disease and eGFR; (E) Celiac disease and CKD; (F) Celiac disease and Urolithiasis; (G) Celiac disease and Acute glomerulonephritis;(H) Celiac disease and NS;(I) Celiac disease and DN;(J) Celiac disease and UACR.

Supplementary Figure S6

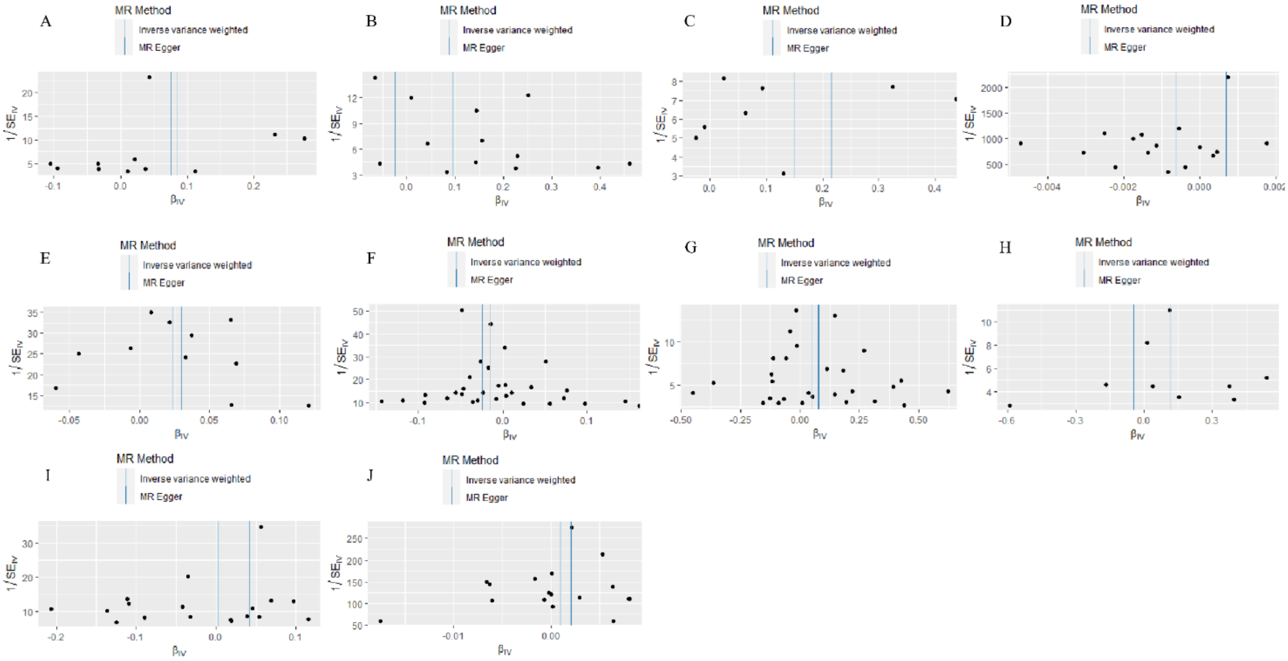

Figure S6 Validation data MR analysis funnel plot: (A) Celiac disease and IgAN; (B) Celiac disease and Chronic glomerulonephritis; (C) Celiac disease and MN; (D) Celiac disease and eGFR; (E) Celiac disease and CKD; (F) Celiac disease and Urolithiasis; (G) Celiac disease and Acute glomerulonephritis;(H) Celiac disease and NS;(I) Celiac disease and DN;(J) Celiac disease and UACR.

Supplementary Figure S7

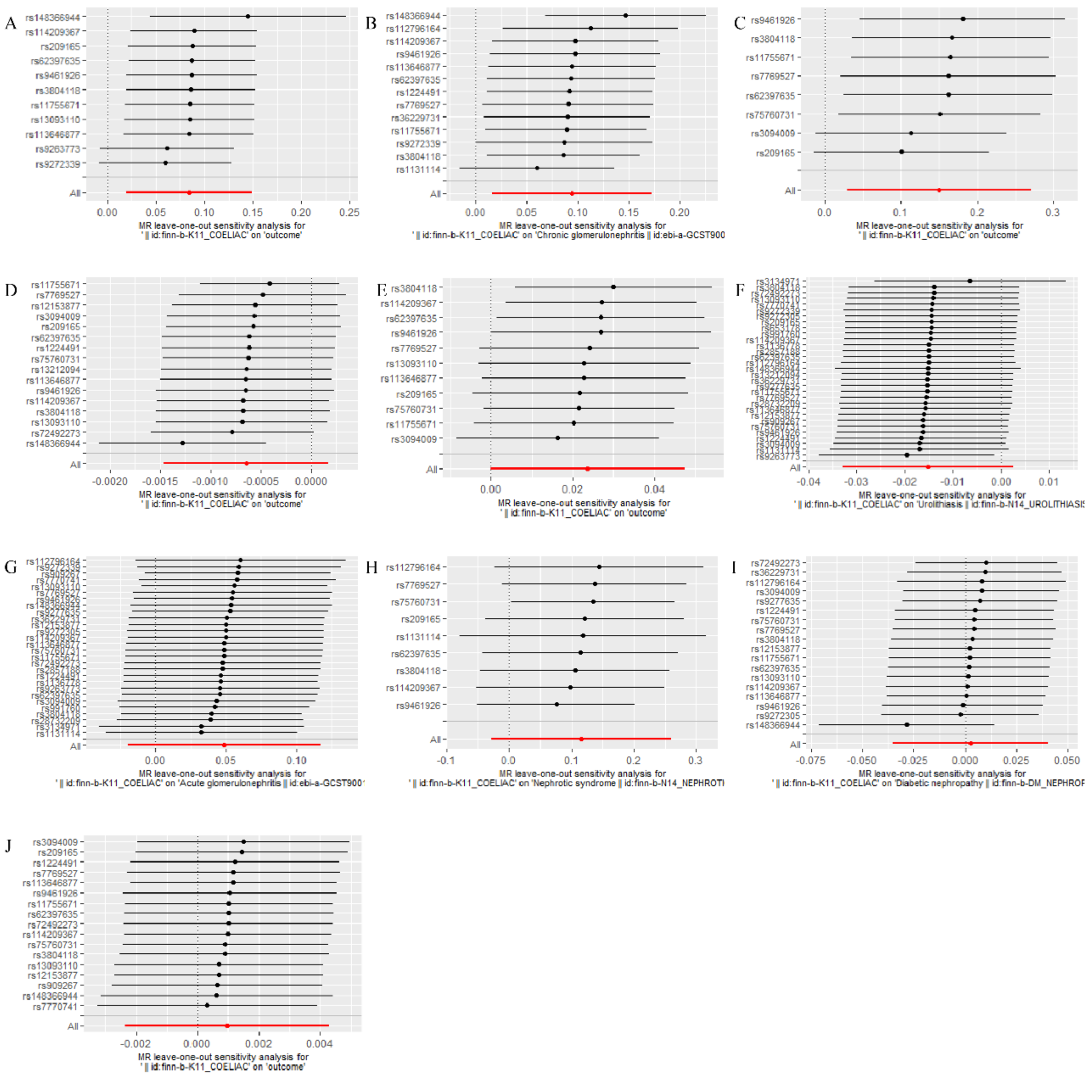

Figure S7 Validation data leave-one-out sensitivity analysis: (A) Celiac disease and IgAN; (B) Celiac disease and Chronic glomerulonephritis; (C) Celiac disease and MN; (D) Celiac disease and eGFR; (E) Celiac disease and CKD; (F) Celiac disease and Urolithiasis; (G) Celiac disease and Acute glomerulonephritis;(H) Celiac disease and NS;(I) Celiac disease and DN;(J) Celiac disease and UACR.

Supplementary Figure S8

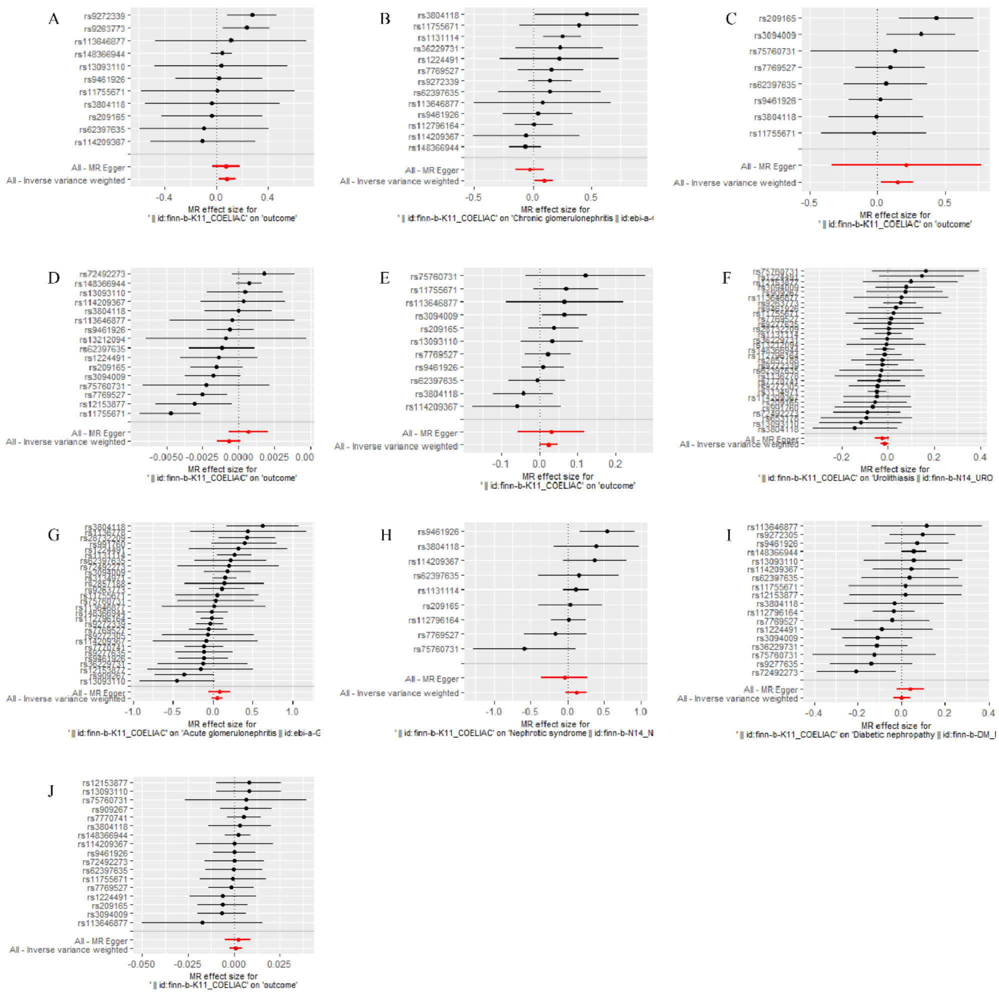

Figure S8 Validation data MR analysis forest plot: (A) Celiac disease and IgAN; (B) Celiac disease and Chronic glomerulonephritis; (C) Celiac disease and MN; (D) Celiac disease and eGFR; (E) Celiac disease and CKD; (F) Celiac disease and Urolithiasis; (G) Celiac disease and Acute glomerulonephritis;(H) Celiac disease and NS;(I) Celiac disease and DN;(J) Celiac disease and UACR.
